# AI literacy and innovative work behavior among Chinese university teachers: indirect associations through AI-TPACK and moderation by organizational inertia

**DOI:** 10.3389/fpsyg.2026.1828350

**Published:** 2026-08-28

**Authors:** Si Xu, Ge Zhang, Wei Xu

**Affiliations:** 1 College of Education, Binzhou Polytechnic University, Binzhou, China; 2 College of Intelligent Science and Engineering, Beijing University of Agriculture, Beijing, China

**Keywords:** AI literacy, AI-TPACK, higher education, innovative work behavior, organizational inertia

## Abstract

**Introduction:**

Artificial intelligence is increasingly integrated into higher education, raising questions about how teachers’ AI-related competencies are associated with innovative work behavior.

**Methods:**

Based on survey data from 510 university teachers in China, this study examined the relationships among artificial intelligence literacy (AIL), artificial intelligence technological pedagogical content knowledge (AI-TPACK), innovative work behavior (IWB), and organizational inertia (OI).

**Results:**

AIL was positively associated with both AI-TPACK and IWB, and a significant indirect association between AIL and IWB through AI-TPACK was identified. OI moderated the associations between AIL and AI-TPACK and between AIL and IWB, with both associations becoming weaker at higher levels of OI. However, the interaction between AI-TPACK and OI in predicting IWB was not statistically significant.

**Discussion:**

These findings highlight the relevance of both teachers’ AI-related competencies and organizational context to innovative work behavior in higher education.

## Introduction

In recent years, generative AI tools such as ChatGPT and other similar platforms have quickly entered university classrooms and daily academic work (Hu et al., 2025). Their presence has changed how information is produced and shared (Ye et al., 2024), and many teachers are now trying to understand what these tools actually mean for their teaching. While some educators welcome these technologies, others feel uncertain about how their roles and responsibilities may shift (Law, 2024). As students also begin to rely on AI-based support for learning, teachers are under growing pressure to rethink their teaching methods and even the way they communicate knowledge (Han and Li, 2026).

Are teachers willing to embrace artificial intelligence in the classroom? This will largely depend on their understanding of a series of problems beyond the technology itself (Biagini, 2025). Compared with related digital or information literacy, artificial intelligence literacy (AIL) uniquely focuses on two levels: one is the cognition of the basic working principle of artificial intelligence, and the other is the examination of the changes brought about by its educational and teaching application (Aji et al., 2026). Therefore, at present, the definition of AIL in academic circles has clearly gone beyond the skill category, and it is a compound accomplishment including ethical standpoint, value judgment and critical use ability (Yuan et al., 2024).

Although AIL has become a hot topic (Kong et al., 2025), there is still a lack of solid empirical evidence about its relationship with university teachers’ innovative behavior. One of the main limitations is that most related studies focus on employees of enterprises, and its conclusions are difficult to be directly transferred to the higher education context because of the obvious distinction between professional background and motivation. This situation highlights a consensus that teachers’ innovation must be understood and studied in their own professional context (Dong et al., 2024). In this study, innovative work behavior (IWB) is conceptualized as a situation-specific construct placed in the teaching environment.

When teachers start to adjust the curriculum, create materials or try new technologies (Prasetyo et al., 2025), they are showing typical innovative work behaviors. This series of behaviors can not only promote teaching reform at the individual level, but also spread to the level of departments or institutions (Brown et al., 2021). Conceptually, IWB is defined as a dynamic process, which starts with problem identification, attempts at methods and finally puts it into practice (Messmann and Mulder, 2012). The key psychological factor that drives teachers to devote themselves to this is their obvious willingness to explore and integrate new resources (Al-Awidi and Al-Furaih, 2023).

Facing the deep integration of AI in education, a key perspective of this study is to examine teachers’ artificial intelligence technological pedagogical content knowledge (AI-TPACK). This concept is born out of the TPACK framework (Mishra and Koehler, 2006), which aims to describe teachers’ professional knowledge of integrating AI knowledge, subject content and teaching methods. It is pointed out that teachers who are more familiar with AI technology and application scenarios also show stronger control ability when introducing AI tools into teaching design (Al-Abdullatif, 2024). Stronger AI literacy has been proved to promote the formation of a more perfect AI-TPACK (Al-Abdullatif, 2024), and the maturity of this professional knowledge indicates the emergence of more innovative work behaviors (Chen et al., 2025).

Although teachers have high AIL, the occurrence of their innovative behavior is not the only determinant (Stumbrienė et al., 2024). Organizational structure plays an equal or even more critical role here. Slow decision-making process and rigid daily operation mode constitute typical organizational inertia (OI), which may directly hinder teachers’ willingness to apply AI (Liu et al., 2024). Therefore, without supportive leadership, flexible policies or sufficient resources, teachers’ unilateral efforts are often difficult to translate into substantive teaching innovation (Lei et al., 2024). This shows that the influence of AIL and AI-TPACK on innovation behavior is subject to organizational environment.

Accordingly, this study developed a theory-informed moderated mediation model to examine whether AIL was indirectly associated with IWB through AI-TPACK and whether these associations varied according to OI. Because all variables were measured concurrently, the model was used to estimate contemporaneous statistical associations rather than establish temporal or causal pathways.

## Literature review

### AI literacy and innovative work behavior

Artificial intelligence is increasingly affecting individuals’ participation in work-related tasks and innovation processes (Zhang et al., 2025). In the educational environment, a higher AIL may enable teachers to better understand and use intelligent tools, thus supporting them to participate in innovative teaching practice (Ji et al., 2025). Innovative work behavior is usually defined as a series of behavior activities carried out by individuals around the generation, promotion and implementation of new ideas in the course of work (Messmann and Mulder, 2014). In the teaching situation, this kind of behavior is embodied in teachers introducing new ideas in teaching design, trying new technologies in classroom practice, and continuously optimizing teaching processes and methods.

However, the relationship between AIL and innovative work behavior is not necessarily direct. Transforming artificial intelligence-related knowledge into practical innovative behavior may depend on a variety of intervention mechanisms and situational conditions (Caroline et al., 2025). Similarly, organizational environment and individual technology perception can promote or limit the behavioral results of artificial intelligence literacy (Dong et al., 2024). Despite these accidental factors, existing studies agree that AIL is an important predictor of job performance and creative results (Huu, 2023). Based on this research direction and considering the innovative work behavior implemented in the teaching environment.

Therefore, the study proposes the following hypothesis:

*H1*: AI literacy is positively associated with innovative work behavior.

### AI literacy and AI-TPACK

AI-TPACK embodies the dynamic ability structure under the background of intelligent education, including the analysis of AI education mechanism, situational evaluation of its teaching adaptability, design of integrated learning activities and iterative optimization ability based on feedback (Al-Abdullatif, 2024). AIL is the key cognitive basis and core component to realize this knowledge integration model, focusing on the educational wisdom of using AI tools strategically to achieve goals in complex teaching situations (Chiu et al., 2024). When teachers lack this literacy, the transformation path from AI technology to teaching practice will be hindered (Oved and Alt, 2025). On the contrary, teachers with high level of AIL usually show a stronger sense of efficiency in technology teaching integration, and can systematically plan the use of AI tools in the whole teaching process (Haroud and Saqri, 2025). This ability ultimately promotes the continuous optimization of its teaching paradigm, promotes the deep integration of technology and curriculum objectives, and effectively stimulates its willingness to carry out continuous teaching experiments and explore innovative teaching practices (Ng et al., 2025).

Thus, the study proposes the following hypothesis:

*H2*: AI literacy is positively associated with AI-TPACK.

### AI-TPACK and innovative work behavior

Teachers’ participation in innovative work is not only driven by their intrinsic innovative motivation, but also depends to a great extent on their knowledge structure in the integration of technology, teaching methods and subject content (Berisha Qehaja, 2025). In this context, AI-TPACK provides a systematic analysis framework for the integration of artificial intelligence and teaching, the core of which is to push teachers from simply using technical tools to deep integration and teaching innovation oriented by teaching objectives (Ning et al., 2024). Teachers with higher level of AI-TPACK usually show stronger adaptability and teaching confidence in the face of teaching reform and technological change (Sun et al., 2023). This ability is helpful to transform its knowledge advantage into practical innovative work behavior. Although the current empirical research has not yet formed a stable relationship between AI-TPACK and innovative work behavior.

Therefore, the study proposes the following hypothesis:

*H3*: AI-TPACK is positively associated with innovative work behavior.

### The mediating role of AI-TPACK

The AI-TPACK developed based on the classic framework of TPACK focuses on how teachers integrate AI into subject content and teaching activities (Balta, 2025). AI-TPACK is conceptualized as a knowledge structure that bridges the gap between understanding and application (Zhai, 2024). Whether teachers can successfully adapt to the AI environment depends on whether technology is absorbed into their professional thinking and usage habits. As the basis of integration, the lack of AIL is likely to hinder the effective use of AI in teaching planning, implementation and evaluation (Salhab, 2024). However, AIL is not generated automatically or directly (Kim, 2023). Only when teachers can successfully combine their understanding of AI with their profound knowledge of subject teaching can their value be fully realized (Kim et al., 2022). It is this integrated knowledge base that enables teachers to go beyond the technical use of AI tools and turn to meaningful applications oriented to teaching intentions, thus creating conditions for the germination of innovative behaviors in daily teaching. Teachers with higher AIL usually show stronger ability to identify and realize the teaching value of AI in their decision-making, which is helpful to form more prudent and creative teaching strategies (Kong and Yang, 2024).

The study proposes the following hypothesis:

*H4*: AI literacy has a significant indirect association with innovative work behavior through AI-TPACK.

### The moderating role of organizational inertia

The degree to which individuals transform AIL into innovative behavior is influenced by their organizational situation (Han et al., 2025). Organizational inertia (OI) is usually manifested in path-dependent operational inertia, rigid decision-making mechanism and high dependence on existing work patterns (Samadi et al., 2024). At the same time, the risk-averse management tendency and cautious attitude towards technological change further aggravate the uncertainty in the process of innovation (Castelo and Ward, 2021). In this situation, even if individuals have a high level of AIL, their innovation potential may be difficult to effectively translate into actual behavior.

On the contrary, in an environment with low organizational inertia, organizations are more likely to support adaptive learning and exploratory practice, thus promoting individuals to use artificial intelligence knowledge, reconstruct work tasks and explore new solutions (Holgersson et al., 2024).

Based on the above analysis, this paper holds that organizational inertia plays a situational adjustment role between artificial intelligence literacy, AI-TPACK and innovative work behavior, which not only affects the process of transforming artificial intelligence literacy into AI-TPACK, but also affects the degree to which related capabilities are further transformed into innovative behavior.

The following hypotheses are proposed:

*H5*: Organizational inertia moderates the association between AI literacy and AI-TPACK.

*H6*: Organizational inertia moderates the association between AI-TPACK and innovative work behavior.

*H7*: Organizational inertia moderates the association between AI literacy and innovative work behavior.

**Figure 1:**
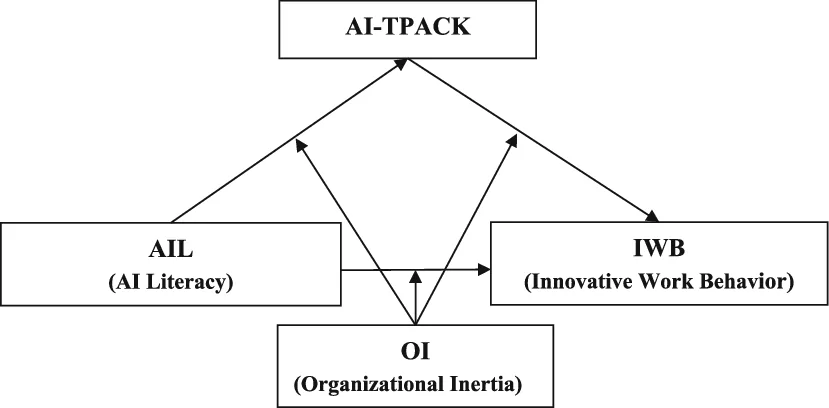
Research model.

The hypothesized moderated mediation model is presented in Figure 1.

## Methods

### Procedures

Before data collection, the study was approved by the ethics review committee of the authors’ affiliated institution. Data were collected in China in July 2025 through a professional online survey platform. A non-probability convenience sampling method was used to recruit university teachers. Participation was entirely voluntary. Before completing the questionnaire, all participants were informed of the study purpose, the confidentiality and anonymity of their responses, and their right to withdraw from the study. Informed consent was obtained from all participants. The collected data were used exclusively for research purposes.

### Participants

Eligible participants were university teachers working in China who had actively participated in at least one AI-supported teaching innovation or teaching reform activity. A screening question was presented at the beginning of the questionnaire: “Have you actively participated in any teaching innovation or reform initiatives involving AI technology?” Respondents who answered “No” were not permitted to proceed to the main questionnaire and were excluded from the subsequent analysis. A non-probability convenience sampling method was employed. Data were collected through Wenjuanxing (Wjx), a professional online survey platform widely used in China. Participants were mainly recruited from universities located in central, eastern, and southeastern China. These regions were selected because they are relatively well developed economically and educationally and have more mature infrastructure and institutional conditions for the application of AI tools in higher education teaching (Dai, 2024). A total of 552 questionnaires were collected. After excluding 42 invalid questionnaires, 510 valid responses were retained, resulting in a valid response rate of 92.39%. The final sample included university teachers with diverse backgrounds in terms of gender, academic discipline, teaching experience, age, and professional title. Because eligibility required prior participation in AI-supported teaching innovation or reform, the sample may include teachers who were relatively more engaged with and receptive to AI than the broader population of university teachers.

### Measurements

All measurement instruments were originally developed in English and were translated into Chinese before data collection. A professional translator first translated the original English items into Chinese. A bilingual research team then compared the translated items with the original versions on an item-by-item basis and revised the wording where necessary to ensure semantic accuracy, conceptual consistency, and clarity in the Chinese higher education context. For the IWB scale, minor contextual adaptations were made to align the item wording with university teaching and teaching innovation while retaining the meanings of the original items. The finalized Chinese version was used in the formal survey. All items were rated on a five-point Likert scale ranging from 1 (*strongly disagree*) to 5 (*strongly agree*).

### AI literacy

AI literacy was measured using the 24-item scale developed by Yuan et al. (2024). The scale comprises three dimensions: cognitive, behavioral, and normative AI literacy. A representative item was “I know what AI can do and what it cannot do yet.” In the present study, Cronbach’s alpha was 0.973 for the overall scale, 0.957 for the cognitive dimension, 0.741 for the behavioral dimension, and 0.914 for the normative dimension. The KMO value was 0.988.

### Innovative work behavior

Innovative work behavior was measured using the 20-item scale developed by Messmann and Mulder (2014). The scale consists of four dimensions: opportunity exploration, idea generation, idea promotion, and reflection. To align the instrument with the higher education teaching context, the wording of the items was contextually adapted. A representative adapted item was “I promote new ideas to colleagues to gain their active support.” Cronbach’s alpha was 0.859 for opportunity exploration, 0.897 for idea generation, 0.910 for idea promotion, 0.818 for reflection, and 0.967 for the overall scale. The KMO value was 0.987.

### AI-TPACK

AI-TPACK was assessed using the 20-item scale developed by Mandal et al. (2025). The instrument comprises four dimensions: TK, GenAI-TPK, GenAI-TCK, and GenAI-XK. A representative item was “I can use GenAI platforms as teaching aids for my subject.” Cronbach’s alpha was 0.902 for TK, 0.884 for GenAI-TPK, 0.889 for GenAI-TCK, 0.861 for GenAI-XK, and 0.969 for the overall scale. The KMO value was 0.986.

### Organizational inertia

Organizational inertia was measured using the nine-item scale adopted from Liu et al. (2024). The scale comprises two dimensions: insight inertia and action inertia. A representative item was “Our university rarely observes changes in the external educational environment.” Cronbach’s alpha was 0.854 for insight inertia, 0.885 for action inertia, and 0.931 for the overall scale. The KMO value was 0.960.

### Data processing

Before testing the hypothesized relationships, CFA was conducted using AMOS to assess the measurement model. Model fit was evaluated using χ^2^/df, CFI, TLI, RMSEA, and SRMR. CR and AVE were calculated to assess construct reliability and convergent validity, respectively. Discriminant validity was evaluated using HTMT. Because all study variables were measured through self-report questionnaires completed by the same sample in a single survey, common method variance was assessed using Harman’s single-factor test.

Data analysis was conducted using SPSS. Descriptive statistics and Pearson correlation analyses were first performed to examine the distributions and associations of the main variables. Multicollinearity was assessed using VIF values for the predictors included in the regression models. The PROCESS macro was used to test the indirect association and moderation models (Hayes and Scharkow, 2013). Specifically, Model 4 was used to examine the indirect association between AIL and IWB through AI-TPACK. Model 59 was selected because the hypothesized model specified OI as a moderator of the association between AIL and AI-TPACK, the association between AI-TPACK and IWB, and the association between AIL and IWB after accounting for AI-TPACK. Simpler moderation models would test these interactions separately and would not estimate the conditional indirect association within a single integrated model. Bias-corrected bootstrap confidence intervals were calculated using 5,000 resamples. Given the cross-sectional design, the mediation analysis was interpreted as an assessment of indirect statistical associations among concurrently measured variables. It did not establish temporal ordering or causal mediation.

## Results

### Measurement model assessment

Before hypothesis testing, CFA was conducted to assess the measurement model. The hypothesized four-factor measurement model demonstrated a good fit to the data: χ^2^ = 2821.356, *df* = 2,549, χ^2^/*df* = 1.107, CFI = 0.990, TLI = 0.990, RMSEA = 0.014, and SRMR = 0.028. The standardized factor loadings ranged from 0.728 to 0.806.

As shown in Table 1, the CR values ranged from 0.932 to 0.973, exceeding the recommended threshold of 0.70. The AVE values ranged from 0.598 to 0.606, with all values exceeding the recommended threshold of 0.50. These results indicated adequate construct reliability and convergent validity. The high alpha coefficients for AIL, IWB, and AI-TPACK may partly reflect their relatively large numbers of items and some overlap in item content. The full scales were retained to preserve multidimensional coverage, although future studies could examine whether shorter versions maintain adequate validity.

**Table 1:** Construct reliability and convergent validity.

Construct	Standardized loading range	Cronbach’s Alpha	CR	AVE
AI literacy	0.752–0.792	0.973	0.973	0.601
Innovative work behavior	0.748–0.797	0.967	0.968	0.598
AI-TPACK	0.755–0.799	0.969	0.969	0.606
Organizational inertia	0.728–0.806	0.931	0.932	0.602

Discriminant validity was assessed using HTMT. As shown in Table 2, the HTMT values ranged from 0.466 to 0.562, all of which were below the conservative threshold of 0.85, indicating satisfactory discriminant validity among the four constructs.

**Table 2:** HTMT ratios for discriminant validity.

Construct	1	2	3	4
AI literacy	—			
Innovative work behavior	0.474	—		
AI-TPACK	0.562	0.531	—	
Organizational inertia	0.481	0.470	0.466	—

### Common method bias assessment

Harman’s single-factor test was conducted to assess potential common method bias. The unrotated principal component analysis extracted four factors with eigenvalues greater than 1. The first factor accounted for 39.407% of the total variance, which was below the commonly used threshold of 40%. Thus, no single factor dominated the variance among the measures, suggesting that serious common method bias was unlikely.

### Descriptive statistics and correlation analysis

Table 3 presents the descriptive statistics and Pearson correlations among the main variables. The mean scores ranged from 2.592 to 3.409, and the standard deviations ranged from 0.915 to 0.952. AIL, IWB, and AI-TPACK had relatively higher mean scores, whereas OI had a lower mean score. All variables were significantly correlated at *p* < 0.01.

**Table 3:** Descriptive statistics and Pearson correlations among key variables (*N* = 510).

Variable	Mean	SD	AIL	IWB	AI-TPACK	OI
AIL	3.393	0.915	1			
IWB	3.409	0.917	0.460**	1		
AI-TPACK	3.405	0.935	0.545**	0.514**	1	
OI	2.592	0.952	−0.458**	−0.446**	−0.443**	1

AIL, AI literacy; IWB, innovative work behavior; AI-TPACK, artificial intelligence technological pedagogical content knowledge; OI, organizational inertia; SD, standard deviation. **p < 0.01.

### Mediation analysis

To examine the indirect association between AIL and IWB through AI-TPACK, PROCESS Model 4 was employed (Table 4). Model 1 showed a significant positive association between AIL and IWB (*β* = 0.461, *p* < 0.001). Model 2 showed a significant positive association between AIL and AI-TPACK (*β* = 0.557, *p* < 0.001). In Model 3, both AIL (*β* = 0.257, p < 0.001) and AI-TPACK (*β* = 0.367, *p* < 0.001) were significantly associated with IWB. These results indicated a significant indirect association between AIL and IWB through AI-TPACK. The VIF values for AIL and AI-TPACK in Model 3 were both 1.422, indicating no evidence of multicollinearity between the predictors. Because all variables were assessed concurrently, these results should not be interpreted as evidence of causal mediation.

**Table 4:** Regression results for the indirect association model (*N* = 510).

Predictor	Model 1 (IWB)		Model 2 (AI-TPACK)		Model 3 (IWB)		VIF
	*β*	t	*β*	t	*β*	t	
AIL	0.461***	11.685	0.557***	14.648	0.257***	5.824	1.422
AI-TPACK					0.367***	8.516	1.422
R^2^	0.212		0.297		0.310		
F	136.542***		214.561***		114.146***		

AIL, AI literacy; AI-TPACK, artificial intelligence technological pedagogical content knowledge; IWB, innovative work behavior; β, standardized coefficient; VIF, variance inflation factor. ***p < 0.001.

Table 5 reports the total, direct, and indirect associations between AIL and IWB through AI-TPACK. After accounting for AI-TPACK, the direct association between AIL and IWB remained statistically significant (*β* = 0.257, 95% CI [0.170, 0.343]). The indirect association through AI-TPACK was also statistically significant (*β* = 0.205, 95% CI [0.150, 0.262]). Thus, AI-TPACK accounted for a significant portion of the statistical association between AIL and IWB. However, because all variables were measured at the same time, this result does not establish temporal or causal mediation.

**Table 5:** Total, direct, and indirect associations of AIL with IWB through AI-TPACK (*N* = 510).

Association						*β*	SE	LLCI	ULCI
Total association between AIL on IWB						0.461	0.039	0.384	0.539
Direct association between AIL on IWB						0.257	0.044	0.170	0.343
Indirect association between AIL and IWB through AI-TPACK						0.205	0.028	0.150	0.262
Independent		Mediator		Dependent					
AIL	**>**	AI-TPACK	**>**	IWB					

AIL, AI literacy; AI-TPACK, artificial intelligence technological pedagogical content knowledge; IWB, innovative work behavior; β, standardized effect; SE, standard error; LLCI, lower limit of the confidence interval; ULCI, upper limit of the confidence interval.

### Testing for the moderated mediation

**Figure 2:**
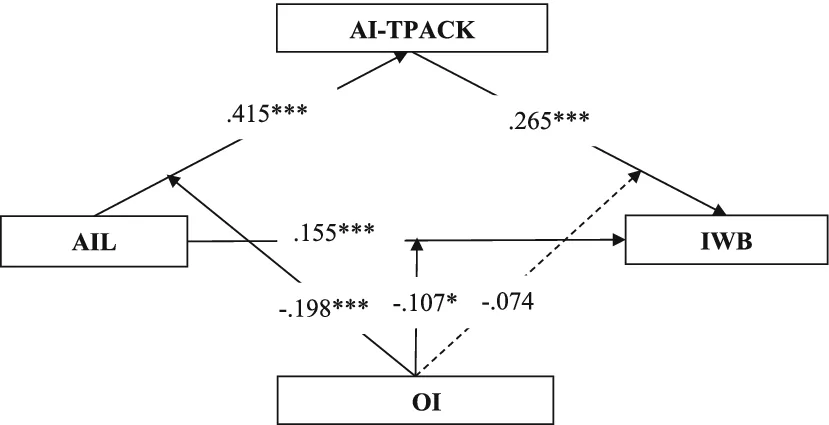
Results of moderated mediation.

PROCESS Model 59 was used to test the moderated mediation model. Multicollinearity diagnostics showed that the VIF values ranged from 1.009 to 1.655, indicating that multicollinearity was not a concern. As shown in Table 6, the interaction between AIL and OI significantly predicted AI-TPACK, indicating that the positive association between AIL and AI-TPACK differed across levels of OI. For IWB, the interaction between AIL and OI was significant, whereas the interaction between AI-TPACK and OI was not significant (Figure 2). These results indicated that the association between AIL and IWB varied across levels of OI, while no evidence was found that the association between AI-TPACK and IWB differed according to OI.

**Table 6:** Regression results of the moderated mediation model (*N* = 510).

Outcome variable	Predictor	*β*	SE	t	95% CI	VIF
AI-TPACK	AIL	0.415***	0.040	10.499	[0.337, 0.493]	1.276
	OI	−0.249***	0.040	−6.286	[−0.327, −0.171]	1.266
	AIL × OI	−0.198***	0.040	−4.902	[−0.277, −0.118]	1.009
R^2^	0.374					
F	100.715***					
IWB	AIL	0.155***	0.042	3.720	[0.073, 0.238]	1.655
	AI-TPACK	0.265***	0.041	6.392	[0.183, 0.346]	1.627
	OI	−0.218***	0.038	−5.734	[−0.293, −0.143]	1.370
	AIL × OI	−0.107*	0.043	−2.468	[−0.193, −0.022]	1.376
	AI-TPACK × OI	−0.074	0.042	−1.768	[−0.157, 0.008]	1.377
R^2^	0.368					
F	58.765***					

AIL, AI literacy; AI-TPACK, artificial intelligence technological pedagogical content knowledge; IWB, innovative work behavior; OI, organizational inertia; β, standardized coefficient; SE, standard error; CI, confidence interval; VIF, variance inflation factor. *p < 0.05, ***p < 0.001.

**Figure 3:**
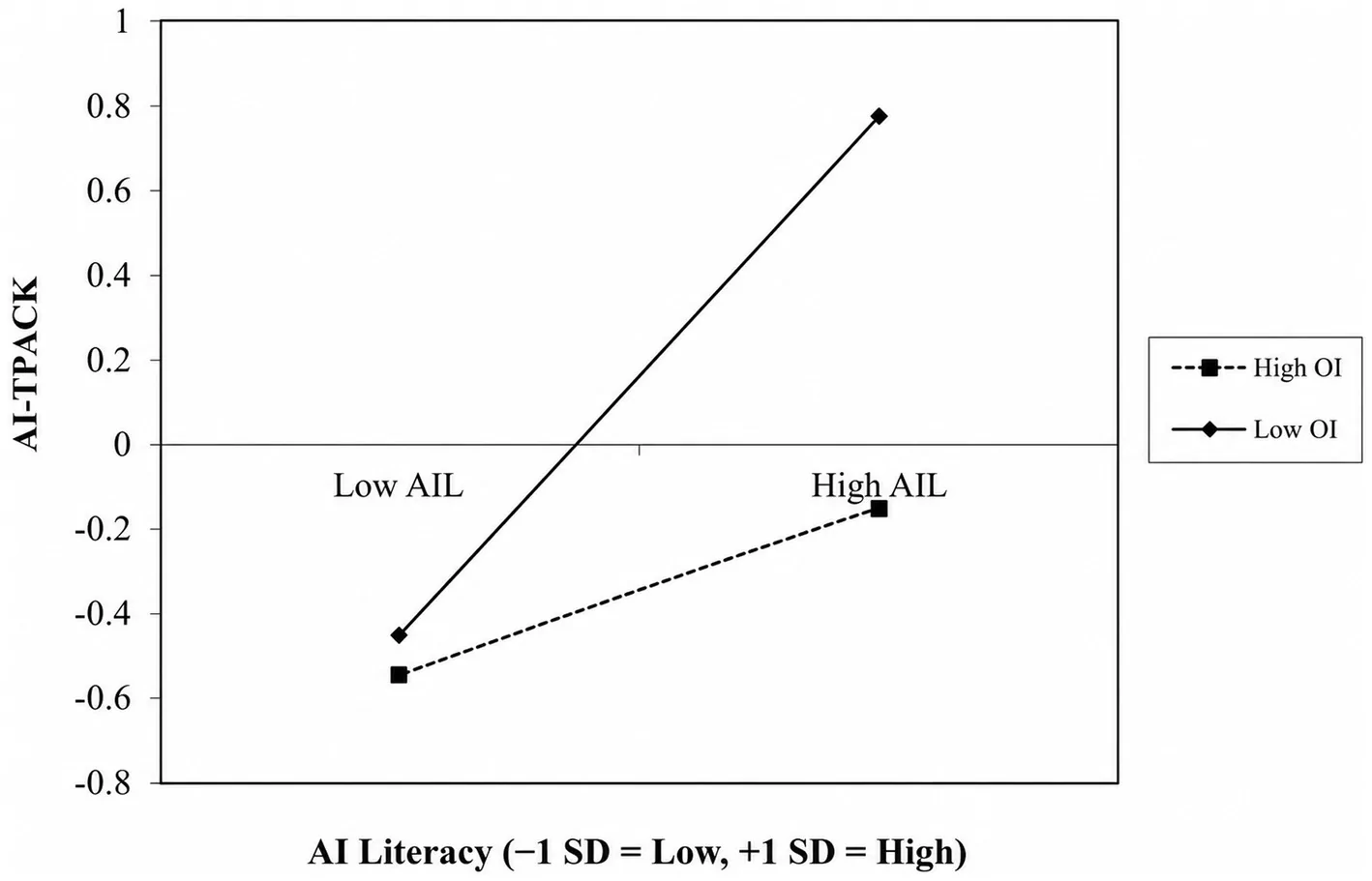
Moderating effect of OI on the relationship between AIL and AI-TPACK.

**Figure 4:**
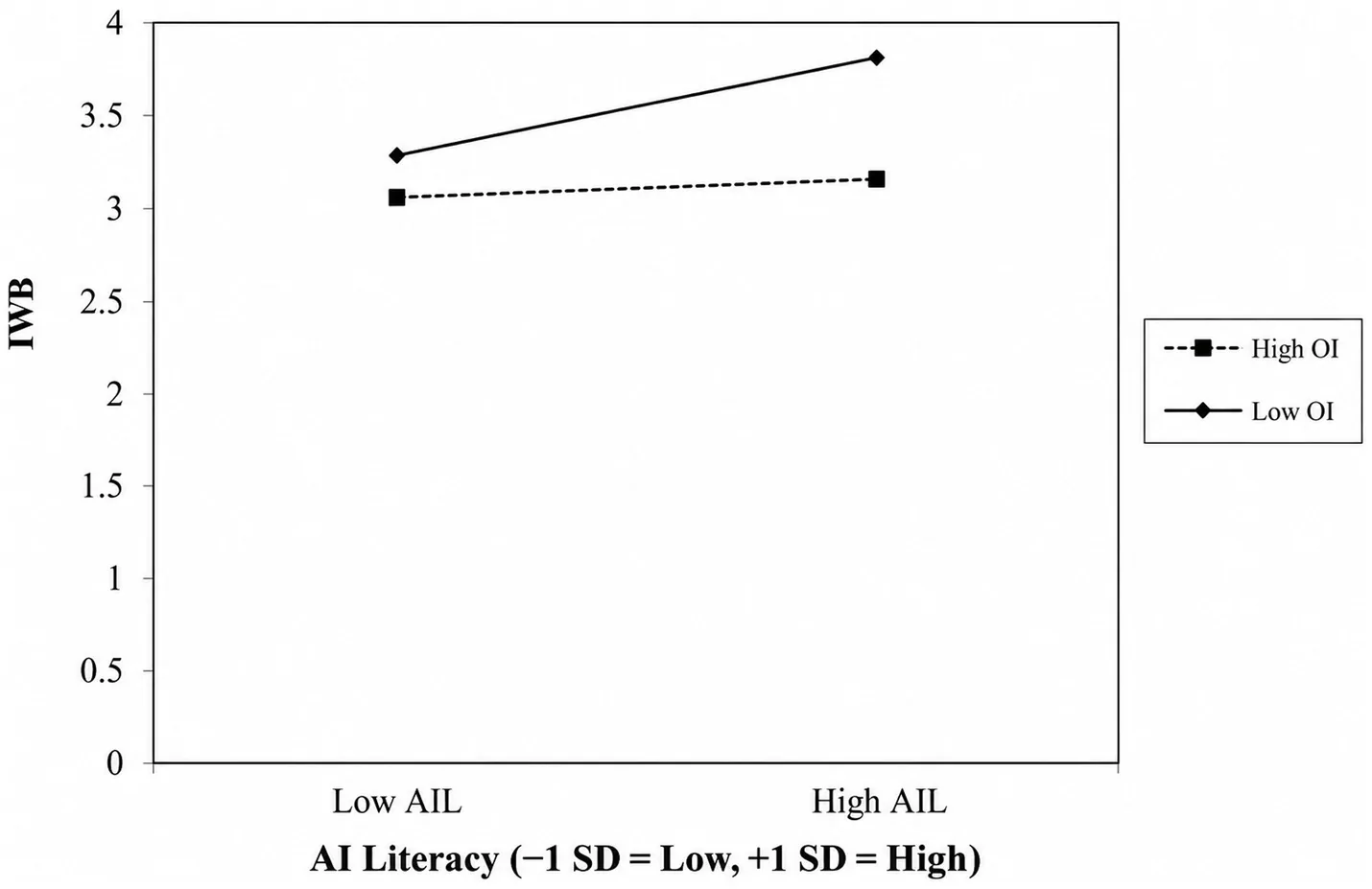
Moderating effect of OI on the relationship between AIL and IWB.

Simple slope analyses are presented in Figures 3, 4. At low levels of OI (−1 SD), AIL was positively associated with both AI-TPACK and IWB, and these associations were stronger than those observed at high levels of OI (+1 SD). At high levels of OI, both associations remained statistically significant but were weaker. These results indicated that the positive associations of AIL with AI-TPACK and IWB were weaker at higher levels of OI.

Table 7 summarizes the hypothesis-testing results. AIL was positively associated with IWB, and a significant indirect association through AI-TPACK was observed. OI moderated the associations between AIL and AI-TPACK and between AIL and IWB, whereas the interaction between AI-TPACK and OI was not statistically significant. All hypotheses except H6 were supported.

**Table 7:** Summary of hypothesis testing.

Hypothesis	Path	Result
H1	AIL → IWB	Supported
H2	AIL → AI-TPACK	Supported
H3	AI-TPACK → IWB	Supported
H4	AIL → AI-TPACK → IWB	Supported
H5	AIL × OI → AI-TPACK	Supported
H6	AI-TPACK × OI → IWB	Not Supported
H7	AIL × OI → IWB	Supported

## Discussion

### Association between AI literacy and innovative work behavior

The findings showed that AIL was positively associated with teachers’ IWB. Teachers reporting higher levels of AIL also tended to report greater engagement in trying new teaching methods and integrating emerging technologies into classroom practice. This finding is consistent with previous research linking digital literacy with educational innovation (Zhang et al., 2025). AIL may therefore be an important individual characteristic associated with teachers’ participation in innovative teaching practices.

### Mediating role of AI-TPACK

The results indicated a significant indirect association between AIL and IWB through AI-TPACK. Teachers reporting higher AIL also tended to report stronger AI-TPACK, which was in turn positively associated with IWB. This pattern is consistent with the TPACK framework proposed by Mishra and Koehler (2006) and related empirical studies (Zhai, 2024). The findings suggest that teachers’ integrated knowledge of AI, pedagogy, and subject content may help explain the statistical association between AI literacy and innovative work behavior.

### Moderating role of organizational inertia

The results showed that OI moderated the associations of AIL with both AI-TPACK and IWB. Specifically, these positive associations were weaker at higher levels of OI. This pattern is consistent with the view that rigid organizational structures and resistance to change may constrain the application of teachers’ technological competencies in teaching practice (Jing et al., 2025; Holgersson et al., 2024; Castelo and Ward, 2021).

By contrast, the interaction between AI-TPACK and OI was not statistically significant, indicating that the association between AI-TPACK and IWB did not vary significantly across levels of OI. One possible explanation is that AI-TPACK reflects teaching-specific integrated knowledge that is more directly connected to instructional practice (Ning et al., 2024). However, this interpretation was not directly tested and should be treated cautiously.

## Conclusion

Among the university teachers included in this study, AIL was positively associated with IWB, and a significant indirect association through AI-TPACK was observed. OI moderated the associations between AIL and AI-TPACK and between AIL and IWB, with both positive associations becoming weaker at higher levels of OI. However, the interaction between AI-TPACK and OI was not statistically significant. These findings highlight the joint relevance of teachers’ individual AI-related competencies and their organizational context to innovative work behavior. Given the cross-sectional and self-report design, these findings represent contemporaneous statistical associations and should not be interpreted as evidence of temporal or causal pathways.

### Practical implications and limitations

Although several associations were statistically significant, their practical importance should be interpreted cautiously. The findings therefore offer preliminary implications for university practice. Universities may consider providing practice-oriented professional development to support teachers’ understanding and integration of AI, while fostering organizational conditions that are open and supportive of innovation. However, given the cross-sectional and correlational design, the study does not establish that professional development or reduced organizational inertia will directly improve AI-TPACK or innovative work behavior. These implications require further validation through longitudinal, experimental, or intervention-based research before specific institutional strategies can be recommended.

There are several limitations to this study. First, all variables were assessed through self-report questionnaires in a single cross-sectional survey, which limits the establishment of temporal ordering and causal mediation and may introduce common method variance. Although Harman’s single-factor test did not identify a dominant single factor, common method variance cannot be completely ruled out. Future studies could employ longitudinal or experimental designs and incorporate data from multiple sources. Second, participants were recruited through convenience sampling from selected regions of China, and all had prior experience in AI-supported teaching innovation or reform. This may have resulted in a relatively motivated and AI-positive sample. Therefore, the findings should be interpreted within the sampled university contexts rather than generalized to all university teachers or higher education institutions. Future studies could use more diverse samples across different cultural, regional, and institutional settings.

## Data Availability

The raw data supporting the conclusions of this article will be made available by the authors, without undue reservation.
